# Supplementary material for: Last Aid Training Online: Participants’ and Facilitators’ Perceptions from a Mixed-Methods Study in Rural Scotland
Source: Healthcare (Basel). 2022 May 16;10(5):918. doi: 10.3390/healthcare10050918 (PMC9141240; doi:10.3390/healthcare10050918)
Supplement: Supplementary file 1 [file healthcare-10-00918-s001.zip › Last Aid Facilitator Participant Interview Topic Guide - File S1.pdf]

CREATING COMPASSIONATE VIRTUAL COMMUNITIES TO SUPPORT PALLIATIVE/END OF LIFE CARE NEEDS IN THE HIGHLANDS

Interview Schedule for Last Aid Training Facilitators

Preparing to Facilitate:

1. Before we start, can you remind us when you participated in the Last Aid training and when you began facilitating these sessions?

2. Please tell us what motivated you to become a facilitator for Last Aid training.

3. What were your expectations as a facilitator?

Facilitation Experiences:

4. How would you describe your understanding of palliative or end of life care needs before participating in Last Aid training?

5. What has changed in your understanding of palliative or end of life care needs since becoming a facilitator?

6. If you could choose 5 words to describe your experience of facilitating the course face to face, what would they be?
    a. What would they be when describing facilitating online?

7. What are the key differences between the two approaches?
    a. Which is the approach you are most comfortable with and why?

8. Overall, what are the top 3 things you have learned from facilitating Last Aid training?

9. What challenges you most as a facilitator of Last Aid training?

Next Steps in Facilitation:

10. What would you like to experience more as a facilitator?

11. If you could make 1 change to facilitating this training course, what would it be?

12. Anything else you would like to share?
