# Supplementary material for: Last Aid Training Online: Participants’ and Facilitators’ Perceptions from a Mixed-Methods Study in Rural Scotland
Source: Healthcare (Basel). 2022 May 16;10(5):918. doi: 10.3390/healthcare10050918 (PMC9141240; doi:10.3390/healthcare10050918)
Supplement: Supplementary file 1 [file healthcare-10-00918-s001.zip › Last Aid Training Participant Survey Questionnaire - File S3.pdf]

Creating Compassionate Virtual Rural Communities to support Palliative / End of Life Care needs in the Highlands

Survey information and consent

Creating Compassionate Virtual Rural Communities to support Palliative / End of Life Care needs in the Highlands is a collaborative project between the Highland Hospice and University of the Highlands and Islands.

The Last Aid training programme delivered by the Highland Hospice aims to promote death literacy and raise awareness on palliative and end of life care issues among the general public.

As you may be aware, this training that has normally been delivered face to face given its sensitive nature is now having to be delivered online due to COVID – 19 restrictions. We are keen to evaluate the impact of this training on your knowledge, understanding, awareness and confidence related to palliative and end of life care issues and decisions.

Your contribution to this research is most welcome and your participation is completely voluntary. **Your results are anonymous, and no identifying features are collected**. The survey should take no more than 30 minutes to complete. You would also be able to complete parts of the survey and resume completion.

Everyone who takes part in the survey will be eligible to participate in interviews that are planned as part of this project. If you wish to be considered as a participant for the interviews, you will be asked to provide your email address at the end of this survey, which will only be used for this purpose and will be deleted once the interview is completed.

This project has been approved by the University Research Ethics Committee at University of the Highlands & Islands.

If you have any questions about the survey, or the wider project, please contact **Dr Leah Macaden who is leading this project** by email at **leah.macaden@uhi.ac.uk.**

1. DEMOGRAPHIC AND SOCIAL VARIABLES

Age

- 18 - 24 years
- 25 - 34 years
- 35 - 44 years
- 45 - 54 years
- 55 - 64 years
- 65 - 79 years
- 80+ years
- Rather not say

Gender

- Female
- Male
- Non-binary (specify)
- Rather not say

What is the highest level of education you have completed?

- National 5 or equivalent
- Highers or equivalent
- Certificate or Diploma
- Undergraduate degree
- Postgraduate degree
- Other (specify)
- Rather not say

Employment status (Please select the most appropriate)

- Employed - full time
- Employed – part time
- Self-employed
- Not working
- Retired

- Student
- Rather not say

Employer

- The NHS
- Social Care
- Third Sector
- Education
- Other
- Rather not say

Residence

- Urban area
- Semi-urban area
- Rural area
- Remote area
- Rather not say

Relationship status (Please select the most appropriate response):

- Married/defacto
- Never married/defacto
- Widowed
- Divorced
- Separated but not divorced
- Partnered but not living together
- Single
- Other (specify)
- Rather not say

Community Connections / Networks:

Support / Network	Yes	No
Neighbours		
Community Groups		
Religious Groups		
Friends		

Do you have a religious or spiritual background?

- Yes (please specify)

- No
- Rather not say

Do you have a current religious or spiritual practice?

- Yes (please specify)
- No
- Rather not say

Reason for choosing to attend the Last Aid Training Programme

- Personal interest in the subject
- COVID – 19
- Long term Condition – Self / Spouse / Others
- Terminal Illness - Self / Spouse / Others
- Related to your area of work

How did you source / access information on the Last Aid Training Programme?

- Friends
- Family
- Colleague
- Email
- Social Media
- Highland Hospice Newsletter / Website

Please Respond Using **Yes / No** to the following about any related work or previous training experience:

1. I do paid work or have done paid work with people at end of life

2. I volunteer or have volunteered with people at end of life

3. I do paid work or have done paid work in a job where I support/ed people through grief and loss

4. I volunteer or have volunteered in a job where I support/ed people through grief and loss

5. I have attended training on helping people with dying, grief or bereavement

<u>The following sections focus on how and if the Last Aid</u> Online Training Programme has influenced your knowledge, understanding and attitudes on Death and Dying and your confidence in caring for someone with End of Life Care needs

1. ATTITUDES TOWARDS DEATH AND DYING

How would you rate your level of agreement with the following statements using the scale below?

1 Strongly Disagree 2 – Disagree 3 – Unsure 4 – Agree 5 – Strongly Agree

	Before Last Aid Training	After Last Aid Training	Not Applicable
We discuss death and dying in my community			
We discuss death and dying in my family			
We discuss death and dying in my workplace			
We discuss death and dying in my church / religious gathering			
I normally avoid the topic of death and dying			
I normally avoid people who are grieving			
I normally tend to avoid conversations about dying and death to avoid upsetting people			
I normally tend to avoid conversations about dying and death because it makes me feel uncomfortable			

2. KNOWLEDGE AND CONFIDENCE ON DEATH AND DYING

2.1 PRACTICAL KNOWLEDGE ABOUT DEATH AND DYING

How would you rate your level of confidence with the following care duties using the scale below?

<u>1 - Not at all confident 2 – Not very confident 3 – Somewhat/reasonably confident 4 – Confident 5 – Very confident</u>

**My confidence in:**	Before Last Aid Training	After Last Aid Training	Not Applicable
Talking about death, dying or grieving to a close friend			
Talking about death, dying or grieving to a child			
Talking to a newly bereaved person about their loss			
Talking to a GP about support at home or in their place of care for a dying person			
Talking to someone who is dying			
Recognising common symptoms experienced by people at the end of life			
Providing comfort care at the end of life (using non-drug methods)			
Feeding a person or assisting them to eat and drink as desired			
Bathing a person			
Lifting a person or assisting to transfer them			
Administering medications to someone who is dying			

2.2 EXPERENTIAL KNOWLEDGE

How comfortable are you with the following aspects using the scale below?

1 – Not at all comfortable 2 – Not very comfortable; 3 – Somewhat/reasonably comfortable 4 – Comfortable; 5 – Very comfortable

**End of Life Care Decision Making & Emotions**	Before Last Aid Training	After Last Aid Training	Not Applicable
Initiating End of Life Care decisions about myself			
Initiating End of Life Care conversations with a family member			
Participating in Advance Care Planning for myself			
Participating in Advance Care Planning for a family member			
Participating in Advance Care Planning for a friend			
Supporting others with death and dying processes			

Prioritising what is important and not important in life			
Being compassionate or kind towards myself			
Empathising with others			
Accessing skills and strategies to cope myself emotionally			
Using my skills and strategies to support others facing similar challenges			

How would you rate your knowledge and understanding on the following aspects (2.3 & 2.4) of End of Life Care using the scale below?

1-None 2-Weak 3-Fair 4-Good 5-Very Good

2.3 FACTUAL KNOWLEDGE

**My Knowledge & Understanding on:**	Before Last Aid Training	After Last Aid Training	Not Applicable
What a Do Not Attempt Cardiopulmonary Resuscitation (DNACPR form) is and how it is used			
What a Treatment Escalation Plan is and how it is used?			
What documents you need to complete in planning for death			
How to navigate the health care system to support a dying person to receive care			
How to access palliative care in my area			
What constitutes quality of end of life			
The law regarding what to do after a death in Scotland			
How to navigate funeral services and options			
The contribution the funeral director can make at end of life			

2.4 KNOWLEDGE ON COMMUNITY RESOURCES

**My Knowledge & Understanding on:**	Before Last Aid Training	After Last Aid Training	Not Applicable
Accessing community support available			
Providing day to day care for the dying person			
Accessing equipment required for care			
Accessing culturally appropriate support			
Accessing emotional support for myself			
Support groups for people with life threatening illnesses			
Support groups for people who are dying			
Support groups for carers for people who are dying			
Support groups for people who are grieving			

3. DECISION MAKING AND INTENTIONS TO SUPPORT END OF LIFE CARE NEEDS

Have you **ever** undertaken or intend to undertake any of the following actions?

**My Decisions / Intentions to support End of Life Care Needs**	Before Last Aid Training	After Last Aid Training	Do not wish to disclose
I have made / plan to make a will			
I have appointed / plan to appoint someone to make decisions regarding finances or property e.g. Power of Attorney			
I have made / plan to have an advance care plan in place			
I have helped / intend to support someone to make plans for end of life e.g. advance care plans, funerals etc			
I have provided/ intend to provide emotional support to a person who is dying			
I have helped/intend to help someone who is supporting a dying person by providing emotional help to carer			
I have supported / intend to support with the hands-on care of a person who is dying			

I have given medications / intend to support with administering medicines to a person who is dying if such a need should arise			
I have kept / intend to keep someone company who is near death			
I have helped care / intend to care for the body of someone who has died			
I was /am interested in facilitating an End of Life support group (Face to Face)			
I was /am interested in facilitating an End of Life support group (Virtually)			
I was / am interested to set up an End of Life Care support group in my local community			

4. COMMENTS / SUGGESTIONS

a. Please complete the following from your experience of attending the Last Aid Training

Modules	Topics	Strengths	Limitations	Suggestions		
				**Keep Content**	**Delete Content**	**Add Information / Content**
1	Death as normal part of life					
2	Planning ahead					
3	Relieving suffering					
4	Final Goodbyes					

b. Suggestions on preferred modes for future delivery of the Last Aid Training Programme

Preferred Mode of Delivery	Yes	No
Face to Face		
Virtual		
Small Group Interaction		

c. **What was your one Take Home Message from this training?**

d. **What would be the one End of Life Care Decision you have thought about most during or since your Last Aid training?**

Would you like to participate in the interviews for this project? **Yes / No**

If your response is Yes, please provide us with your email address in the box below which will be used only for the purpose of contacting you to arrange a convenient date and time for your interview.
