# Supplementary material for: Last Aid Training Online: Participants’ and Facilitators’ Perceptions from a Mixed-Methods Study in Rural Scotland
Source: Healthcare (Basel). 2022 May 16;10(5):918. doi: 10.3390/healthcare10050918 (PMC9141240; doi:10.3390/healthcare10050918)
Supplement: Supplementary file 1 [file healthcare-10-00918-s001.zip › Last Aid Training Participant Interview Topic Guide - File S2.pdf]

CREATING COMPASSIONATE VIRTUAL COMMUNITIES TO SUPPORT PALLIATIVE/END OF LIFE CARE NEEDS IN THE HIGHLANDS

<u>Interview Schedule for Last Aid Training Participants</u>

1. Before we start, can you remind us when you participated in the Last Aid training?

<u>Training Preparations:</u>

2. Please tell us what motivated you to participate in the Last Aid training course.

3. What were your expectations of the training before you started?

<u>Training Experiences:</u>

4. If you could choose 5 words to describe your experience of the training, what would they be?

5. How would you describe your understanding of palliative or end of life care needs before the Last Aid training?

6. What has changed in your understanding of palliative or end of life care needs since taking the training for you personally?

7. How has this training influenced your role in promoting these discussions

   a. within in your family?
   b. within your community / place of work?

8. How has this training influenced your participation in End of Life Care decisions
   a. within the family?

b. within your community / place of work?

9. How have you applied what you learned in the training in other ways in your everyday life?

10. Overall, what would be the top 3 things you took away from your training?

Training Format:

11. If you were to design a course such as this, what format would you adopt?

12. If you were the course facilitator, how would you facilitate this course?

13. Any comments / feedback on the links that were provided for additional resources on the subject

14. Anything else you would like to share?
